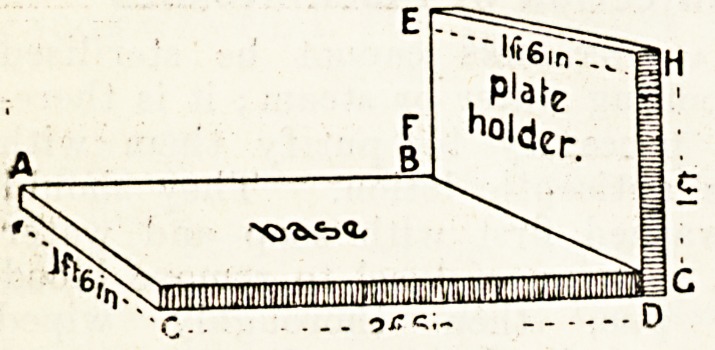# The Institutional Worker

**Published:** 1913-09-27

**Authors:** 


THE
### INSTITUTIONAL WORKER
Being a Special Supplement to "The Hospital"

### OUR BUREAU OF INFORMATION.

### Rules for Correspondents.

1. Every letter must be accompanied by the coupon to be cut from the back cover (inside page) of The Hospital, current issue, and must contain the name and address of the correspondent with pseudonym for publication if desired. Replies by post cannot be given, save under exceptional circumstances at the Editor's discretion.

2. Letters from Approved Homes in reply to special needs published in the Bureau should state terms and full particulars, and be sent prepaid under cover to the Editor of the Bureau with name written across coupon for identification.

3. Proprietors of Homes which have not yet been entered on the List of Approved Homes, but have spare accommodation likely to suit special needs, are invited to write for an application form for registration. The fee for registration, which includes two announcements of the Home in the Bureau and other privileges, is 10s.

4. All communications to be addressed to the Editor of The Hospital, 28 Southampton Street, Strand, London, W.C., and marked "Bureau of Information."

### INSTITUTIONAL FACTS AND FIGURES.

### SOME HINTS ON DISINFECTION.

### Ward Kitchen Disinfectors.

It is not an uncommon practice in hospitals and other institutions for cases to be marked "Keep separate." This may be necessary for a variety of reasons which do not concern us here. It is, in fact, a sort of bed isolation such as is often practised in fever hospitals. The ordinary method of carrying this out is the rough-and-ready one of marking all the china and cutlery and other articles used by the patient. Bits of strapping used to be frequently used for the purpose. The things were then washed up more or less separately, and then, of course, served out again to that particular patient. Such a method is at best of very limited utility. What is now aimed at is a ready means of ensuring that all the utensils—cups, knives, forks, spoons, spatulas, etc.—in the whole ward shall be clean and aseptic so that it does not matter which patient gets a particular article. This is the only safe method, and naturally far preferable even if all the people in the ward were quite free from any contagion. Even carefully washed articles are bound to come in contact with water contaminated already and cloths which have been used for other people's utensils.

The Apparatus Required.

All that is necessary is a convenient sized tank with a steam jacket. This will be connected with the steam supply of the institution. Such a contrivance on a small scale is now frequently to be met with in the shape of an instrument steriliser. A larger affair could easily be fixed by the engineer in the ward kitchen alongside the sink. It should be large enough to contain all the plates, dishes, knives, and forks used by the patients at one time. These are to be dropped in just as they are when the "leavings" have been scraped off into the refuse bin. The steam is turned on, and the whole lot allowed to remain till the water has been boiling for ten or fifteen minutes. Then the water is allowed to cool down to about 100° F. and the contents removed and finished off in the sink in the usual way. A large wire tray will be found a great convenience, for on this the crockery, etc., can be lifted out before the water has cooled, and then the articles can be dealt with at an earlier stage. Some trouble may be expected from the grease which is inevitably present, but by having a large bore tap communicating through the jacket with the inside of the tank or cauldron at a level of about 6 inches below the top of the water, this amount of water with the supernatant grease can be run off. It will not be necessary to completely empty the tank after every batch of dishes, etc., but for this purpose a tap is provided underneath the tank in the centre of the concave bottom. Such tanks are simply replicas of the ward steam sterilisers now so frequently fitted in modern fever hospitals for the sterilising of spatulas used in routine examinations of throats. A well designed and strongly constructed model is made by James Slater and Co., 50 Wells Street, W.C., but other firms will supply these tanks at short notice.

### Disinfection of Bed-Pans.

There is no doubt but that boiling water if properly employed is the most valuable and practical disinfectant. Its range of usefulness is very great. Boiling water is particularly valuable for the disinfection of discharges, for there is no other agent which is so sure. If steam is not available, some simple and inexpensive gas or oil stove may be utilised. A point that is not sufficiently borne in mind is that for the disinfection of intestinal discharges the receptacle to contain the water should be made sufficiently large to receive the bed-pan and its contents just as it comes from the patient, in order that both may be disinfected at the same time. This is a very important detail, as it is in the handling and attempted disinfection of the bed-pan after the discharge is thrown out which is undoubtedly a source of infection to the nurse. A receptacle of this kind also provides an efficient means of disinfecting other infected material such as sputum in spitting cups. The addition of a small quantity of permanganate of potash will be useful in preventing any unpleasant odour arising during the disinfection. The steam escaping may be led out of the room in some simple way to the outer air. One apparatus now in use consists of a copper receptacle which holds six bed-pans. It is heated by gas, and the outflow is connected with the sewer, and from the top is a spout connected by a flexible tube which conducts the steam out of the window. The bed-pan and its contents are left for fifteen minutes after the water is actually boiling. This apparatus is practical and sure, and the nurse is exposed to the minimum risk of infection.

### Disinfection of Mackintoshes

Mackintoshes cannot be sterilised by boiling water or steam; it is therefore necessary to purify them with some antiseptic lotion. They should be washed first with soap and water or a solution of lysol to remove blood and pus, then thoroughly wiped with carbolic lotion and hung up to dry. Care should be taken not to fold them while wet, for the surfaces may adhere. A very satisfactory substitute for mackintosh is *batiste*, which can be sterilised by steam without damage. It should be sponged with cold water and dried, when it will be ready to place in the steriliser. The material should not be immersed in water, as this soon renders it soft and useless.

### Questions for September.

**No. 1 Annual Cleaning.**

**What must be the essential details involved in the annual cleaning and painting, and what is the best method of supervising the work? [Detailed information as to the type and relative cost of paints and enamels found by experience to be most suitable should be given, and any suggestion on the best way to make the supervision effective will be favourably regarded in selecting the replies.]**

**No. 2. X-Ray Apparatus**

**Give particulars of the X-ray apparatus in use at your institution, the names of makers and cost of installation. State the number of each class of cases dealt with, and give a detailed account of the annual cost of maintenance.**

RULES.

The following rules must be observed:—

1. Contributions must be written on one side of the paper. They must bear the name and address of the sender and be accompanied by coupon to be cut from the back cover (inside page) of the current issue of THE HOSPITAL. A pseudonym must be chosen if the name is not to be published.
2. They must be addressed to the Editor of THE HOSPITAL, 28 & 29 Southampton Street, Strand, London, W.C., so as to reach him before the end of the month, and must be marked in the left-hand corner "Facts and Figures."

**A minimum payment of Half a Guinea will be made for each published answer.**

### Home-Made X-Ray Appliance.

WE publish a description of a simple "home-made" appliance sent to us by an institutional worker, which he states has proved useful at his institution as an aid to radiographing limbs in a lateral position in the absence of the usual conveniences provided in a fully equipped *x*-ray room. A piece of wood, say, 2 feet 6 inches by 1 foot 6 inches by 1 inch, is screwed or dovetailed at right angles to another piece, say, 1 foot 6 inches by 1 foot by 1 inch, as in the figure, so that the piece of wood ABCD forms a base upon which the limb is rested, while EFGH is a plate-holder to which the plate to be exposed is attached by means of elastic bands. The method of using the appliance is as follows:—Supposing that a side-view radiograph of the knee is required, the base would be slipped under the leg in such a way as to bring the knee parallel to the plate-holder and in contact with it. In the case of a radiograph of the foot other than a side view, which would be dealt with as described, the base would be placed under the heel in such a position as to bring the plate-holder into contact with the sole of the foot. The advantage claimed for the appliance is that the limb and plate are held in truer relationship than would be possible if the plate were merely packed up with sandbags against the limb; it will also avoid movement when taking the radiograph.

### THE EDITOR'S LETTER-BOX.

### *THE SICK AND IN NEED.*

#### Helpers Wanted for London Creche.

IN connection with the Mothers' Dinners Scheme of the National League of Service a small crèche has been opened near King's Cross, where the mothers leave their babies and young children during the hour when they are eating their dinner. The National League of Service is not able to provide any funds towards the expenses of this crèche or to assist it in any way, as it has more than enough to do to carry on its own legitimate work. The expenses are, therefore, borne by one or two ladies who realise the value to the mothers of this freedom from the care of their children during one hour in the day and the help it is to them to be able to have their dinner quietly and untroubled. The change is also beneficial to the children. Unfortunately, unless some voluntary helpers can be found to undertake to look after the children for one hour each day this beneficent scheme must be abandoned and the crèche closed, as it is impossible to carry it on without voluntary aid of this kind. No gift of money is asked, but only the precious gift of service, and that only for one hour (2 P.M. to 3 P.M.) each day. If six ladies were to offer each to give one hour's service each day in this way the crèche could be continued; otherwise it must be closed. Offers should be addressed to G. L., care of the Editor, THE HOSPITAL Bureau of Information, 28 & 29 Southampton Street, Strand, W.C.

#### Home for Defective Boys.

APART from the provision made by the Poor-Law authorities, homes where crippled boys, suffering from disorders such as you mention, can be received for education, are by no means easy to find. At certain institutions privilege of admission is limited to local areas, and at others the age-limit would preclude the admission of the cases in question. We are prosecuting inquiries, and will advise you in a later issue. Meanwhile it would be helpful to us if you will give us a brief history of the cases, and state what payment could be made towards maintenance. —G. H. H.

#### A Need Supplied.

WE are glad to be able to announce that one of our readers has kindly offered to provide a bath-chair for the poor woman suffering from arthritic rheumatism on whose behalf we issued an appeal in these columns.

**The Editor is prepared to make known without charge the needs of any who may be sick or in difficulties, and to guide them in making choice of Homes for treatment or convalescence. Reduced terms can often be arranged with Homes on the Approved List for those unable to pay full fees. No Home is included in the Approved List without medical and other testimony to its good management. Queries are specially invited from those who are engaged in any kind of philanthropical work.**

### *EMPLOYMENT AND TRAINING.*

#### Hospital Almoner.

YOU cannot obtain a position as hospital almoner without a special training for the work, for the duties necessitate a knowledge of the character and needs of the sick poor, and of the methods of securing for them the assistance of charitable agencies. The Hospital Almoners Council undertakes the training of suitable candidates for a fee of £5 5s. Write to the Secretary, Hospital Almoners Council, Denison House, Vauxhall Bridge Road, London, S.W. An instructive article upon the duties of a hospital almoner appeared in THE HOSPITAL on May 18, 1912.—M. B.

#### Tuberculosis Nursing.

WE do not give postal replies. A coupon should be enclosed with each inquiry; please send one. There is no general rule at present as to the special qualifications of a nurse for tuberculosis work. Committees may, of course, prefer candidates who have had experience in a sanatorium or in a hospital for diseases of the chest. A training school for nurses for instruction in nursing patients suffering from pulmonary tuberculosis has recently been established at the Royal Hospital for Diseases of the Chest, City Road, London. A course of lectures is given and certificates are granted to those who pass a satisfactory examination. Similar courses of lectures are arranged at other chest hospitals.—S. S. B.

### *ACKNOWLEDGMENTS.*

**Helpful.**—We should be glad to hear whether you were successful in obtaining temporary accommodation for your sister at one of the homes suggested in these columns.

**M. E. A. M.**—We have duly forwarded your communication. We regret to hear that you have received so few replies. We make a point of requesting that the courtesy of a reply be extended to the principals of approved homes, though we are unable to enforce it.

**C. D. B.**—We thank you for your letter supplying us with the information we asked for in our last issue respecting the crippled nurse who seeks some suitable occupation and the necessary training. We are making the necessary inquiries, and will advise you in a later issue as to what course may be possible in the direction she wishes. If we find it necessary we will appeal to our readers.

The Editor is indebted for testimonials received in response to inquiries from Mrs. W. Robinson, Dr. Heptinstall, Mr. A. Austin-Davison, Mrs. Chalmers Mitchell, Dr. Eric Pritchard, and Mrs. Percy Smith.

28 & 29 Southampton Street, Strand, London, W.C.,**

### Editor's Notices.

### Contributions :

Contributions should be written, or preferably typed, on one side of the paper only, and all articles sent in are accepted upon the distinct understanding that they are forwarded to THE HOSPITAL only.

The Editor cannot undertake to return MSS. not used, but when a stamped directed envelope is enclosed the MSS. may be returned if a special request is made.

Accepted articles and paragraphs of news will be paid for after publication at the scale rate.

### Address :

To prevent delay all contributions and letters on editorial business must be addressed exclusively to the Editor, "The Hospital," 29 Southampton Street, Strand, London, W.C. It is important that this regulation shall be strictly observed.

### Correspondence :

Correspondence on all subjects is invited. The name and address of correspondents must be given as a guarantee of good faith, but not necessarily for publication.

### Administrative Medicine, Research and Items of News :

Special terms will be paid for approved articles on subjects in this wide field by experts who have made some section of it their special study and interest. We wish to give prominence to every movement tending to advance accuracy of diagnosis, efficiency in treatment, and the development of modern methods to eradicate disease and promote the welfare of the suffering.

### A Bureau of Information :

We invite inquiries and applications for information and help in providing for that numerous class of sufferers who require special care or house-room which they cannot obtain in their own homes or secure for themselves. W cannot, however, prescribe, or recommend practitioner

NEARLY

**400 NEW WORDS**

have been added to the

**NEW EDITION**

of

### The Nurse's Pronouncing Dictionary

**OF MEDICAL TERMS AND NURSING TREATMENT**

Compiled by **HONNOR MORTEN.**

Eighth Edition, thoroughly Revised and much Enlarged.

Edited by **MARY I. BURDETT.**

The new issue of this well-known Dictionary is the **most complete work** of the kind published. It has been completely revised and some hundreds of new words have been added, consequently the utmost reliance can be placed in the information it contains.

To be up-to-date you should GET A COPY TO-DAY.

**Price, in cloth, 2/- post free; in leather, 2/6 net, by post 2/8.**

*Of all Booksellers, or of—*
**The Scientific Press, Ld.**
*(The Nurses' Publishers),*
**28/29 Southampton Street, STRAND, LONDON, W.C.**

### HANDBOOKS
### For TRAINED WORKERS

POCKET GUIDE SP SERIES

**1/- net. 1/1 Post free.**

**Bound in Limp Cloth.**
**Suitable Size for the Pocket.**

The works which comprise the S.P. Pocket Guide Series are intended for ready reference, consequently the aim of the Publishers has been to make them as concise and lucid as possible. Each book is written by an expert on the subject of which it treats, and the utmost reliance therefore can be placed in the information it imparts.

**Essentials of Fever Nursing.** By LYTTON MAITLAND, M.D. (Lond.), M.B., B.S., D.P.H. (Camb.).

**Manual and Atlas of Swedish Exercises.** *(With over 60 Illustrations.)* By THOMAS D. LUKE, M.D., F.R.C.S.

**Bandaging Made Easy.** *(Illustrated with over 90 Diagrams.)* By M. HOSKING, Sister-in-Charge, Tredegar House, Bow, E.

**How to Write and Read Prescriptions.** By LYTTON MAITLAND, M.D. (Lond.), M.B., B.S., D.P.H. (Camb.).

**Principal Drugs and their Uses.** By A PHARMACIST.

**Asepsis and How to Secure It.** By H. W. CARSON, F.R.C.S.

**Treatment after Operations.** *Rules for Nursing after General and Special Operations.* By MARY WILES.

**The Nurse's Duties before and during Operations.** By MARGARET FOX, Matron, Prince of Wales's General Hospital, Tottenham.

**Other works, in amplification of this Series, will be announced in due course.**

*Published by*
**The SCIENTIFIC PRESS, Ltd.**
**28/29 Southampton Street, STRAND, LONDON, W.C.**

By Post **5/4**

**It is possible to secure "the most up-to-date and Complete Guide to Nursing that has yet been written."**

*Vide THE NURSING MIRROR.*

### A HANDBOOK FOR NURSES

**With Examination Questions based upon the contents of the Chapters.**

**By J. K. WATSON, M.D.Edin., C.M.**

**5th Edition, Entirely Revised, and Enlarged to 544 pages**

The title of this book very inadequately describes its scope. It would be more accurately entitled "*THE* Handbook for Nurses," since the information it contains renders it of real and genuine service to the modern Nurse. The fact that **31,500** copies of former editions have been sold is indisputable evidence of its value. The work forms practically an Encyclopædia of Medical, Surgical and Monthly Nursing, and there are few, if any, Nursing details that do not find representation in its pages. If you have not seen the New Edition we would strongly advise you to take the earliest opportunity of doing so.

It is obtainable of all Booksellers, or direct from . . . .

**THE NURSES' PUBLISHERS,**

**28/29 SOUTHAMPTON STREET, STRAND - LONDON, W.C.**

## Figures and Tables

**Figure f1:**